# Homosexual Obsessive–compulsive Disorder Comorbid with Bipolar Disorder: A Rare Case report

**DOI:** 10.1192/j.eurpsy.2023.1962

**Published:** 2023-07-19

**Authors:** M. Gardabbou, R. Feki, N. Smaoui, I. Gassara, S. Omri, M. Maalej Bouali, N. Charfi, J. Ben Thabet, L. Zouari, M. Maalej

**Affiliations:** psychiatry C department, Hedi Chaker, Sfax, Tunisia

## Abstract

**Introduction:**

While bipolar disorder–obsessive compulsive disorder overlap is quite common, sexuality remains a largely unexplored area of this clinical entity.

**Objectives:**

Illustrate through a clinical vignette the case of a patient with diagnosed homosexual obsessive –compulsive disorder (OCD) comorbid with bipolar disorder (BD).

**Methods:**

The clinical case report was prepared through the review of the patient´s clinical record.

**Results:**

We report a rare case of a 22 year-old man who was diagnosed with Homosexual Obsessive–compulsive Disorder comorbid with Bipolar Disorder, admitted to our department for a suicide attempt. He came from a religious and conservative background and suffered from intrusive, unwanted mental images of homosexual behaviour since the age of 17. He presented periods of remission from his obsessive thoughts, while showing signs of elevated mood, talkativeness, restlessness, agitation and hyperactivity that would last for a few days, with recrudescence of obsessive and depressive symptoms again afterwards. The present case showed a significant reduction in depressive symptoms and in the impact of his obsessive intrusive thoughts after prescription of Risperidone and Sodium Valproate along with Exposure and Response Prevention Therapy conducted over a period of 6 weeks.

**Conclusions:**

The homosexual OCD comorbid with bipolar disorder can cause important distress and impairment and severely impact a person’s life in multifaceted ways. Correct diagnosis, adequate medication and psychotherapy provide the effective treatment.

**Disclosure of Interest:**

None Declared

